# Effects of metformin on metabolism of white and brown adipose tissue in obese C57BL/6J mice

**DOI:** 10.1186/s13098-019-0490-2

**Published:** 2019-11-27

**Authors:** Tao Yuan, Juan Li, Wei-Gang Zhao, Wei Sun, Shuai-Nan Liu, Quan Liu, Yong Fu, Zhu-Fang Shen

**Affiliations:** 1 Department of Endocrinology, Key Laboratory of Endocrinology of The National Health and Family Planning Commission, Peking Union Medical College Hospital, Chinese Academy of Medical Science and Peking Union Medical College, Beijing, China; 2 Department of Endocrinology, Beijing Tiantan Hospital, Capital Medical University, Beijing, China; 3 Core Facility of Instrument, Institute of Basic Medical Sciences, Chinese Academy of Medical Sciences/School of Basic Medicine, Peking Union Medical College, Beijing, China; 4 State Key Laboratory of Bioactive Substances and Functions of Natural Medicines, Institute of Materia Medica, Diabetes Research Center of Chinese Academy of Medical Sciences, Chinese Academy of Medical Sciences and Peking Union Medical College, Beijing, China

**Keywords:** Obesity, Adipose tissue, Proteomics, Metformin

## Abstract

**Background:**

To investigate effects of metformin on the regulation of proteins of white adipose tissue (WAT) and brown adipose tissue (BAT) in obesity and explore the underlying mechanisms on energy metabolism.

**Methods:**

C57BL/6J mice were fed with normal diet (ND, n = 6) or high-fat diet (HFD, n = 12) for 22 weeks. HFD-induced obese mice were treated with metformin (MET, n = 6). After treatment for 8 weeks, oral glucose tolerance test (OGTT) and hyperinsulinemic–euglycemic clamp were performed to evaluate the improvement of glucose tolerance and insulin sensitivity. Protein expressions of WAT and BAT in mice among ND, HFD, and MET group were identified and quantified with isobaric tag for relative and absolute quantification (iTRAQ) coupled with 2D LC–MS/MS. The results were analyzed by MASCOT, Scaffold and IPA.

**Results:**

The glucose infusion rate in MET group was increased significantly compared with HFD group. We identified 4388 and 3486 proteins in WAT and BAT, respectively. As compared MET to HFD, differential expressed proteins in WAT and BAT were mainly assigned to the pathways of EIF2 signaling and mitochondrial dysfunction, respectively. In the pathways, CPT1a in WAT, CPT1b and CPT2 in BAT were down-regulated by metformin significantly.

**Conclusions:**

Metformin improved the body weight and insulin sensitivity of obese mice. Meanwhile, metformin might ameliorate endoplasmic reticulum stress in WAT, and affect fatty acid metabolism in WAT and BAT. CPT1 might be a potential target of metformin in WAT and BAT.

## Background

Obesity is a complicated disorder that characterized mainly by excessive adipose tissue accumulation, and as a result of an imbalance between energy intake and energy expenditure. White adipose tissue (WAT) and brown adipose tissue (BAT) are two main types of adipose tissue in mammals. WAT, especially visceral adiposity, is strongly associated with metabolic disorders. Adipokines, secreted by WAT, function as pro-inflammatory or anti-inflammatory cytokines. Some promote insulin resistance and inflammation; others improve insulin sensitivity in obesity. Thus, abnormal secretion of adipokines since the dysfunction of WAT can lead to insulin resistance in obesity and its related complications [1]. Different from WAT, BAT has small lipid droplets and is rich in mitochondria. Uncoupling protein 1 (UCP-1), which exclusively expressed in the inner membrane of the mitochondria in brown adipocytes, mediates energy expenditure and heat production. Human studies predicted that BAT might be a promising target for the treatment of obesity [2].

Metformin is a classical anti-diabetes agent,that not only reduces blood glucose and cardiovascular risk, but also induces weight loss and improves insulin resistance [3, 4]. It is reported that metformin can cross the blood brain barrier, inhibit the activity of adenosine monophosphate-activated kinase and decrease neuropeptide-Y (NPY), thereby reduce food intake [5]. Metformin potentially raise leptin receptor expression and improve leptin response [6]. In addition, metformin may elevate the level of glucagon-like peptide-1 (GLP-1) and enhance satiation signals [7]. In recent years, some researches supported that BAT maybe a target of metformin [8]. Lipid droplet content of BAT was reduced by metformin in mice [9]. Metabolic disorder was ameliorated by metformin via induction of fibroblast growth factor 21 (FGF21) in adipose tissue [10]. However, effects of metformin on lipid metabolism in WAT and BAT have not yet been deciphered.

Our previous research indicated that high-fat diet (HFD) might increase the apoptosis of brown adipocytes by up-regulated apoptosis-inducing factor 1 (AIF1). Besides, HFD also activated fatty acid β-oxidation and enhanced compensatory energy consuming through up-regulating carnitine O-palmitoyl transferase 2 (CPT2) and UCP1 in BAT, respectively. Thus, we aimed to examine the effect of metformin on WAT and BAT in HFD-induced obese mice and explore potential targets of metformin and underlying mechanisms of energy metabolism. Using isobaric tag for relative and absolute quantification (iTRAQ) coupled with two dimensional (2D)–LC–MS/MS, we performed comparative proteome analysis of WAT and BAT among three groups including, normal diet (ND), HFD, and metformin intervention (MET) group. The main advantage of iTRAQ is high throughput and high stability; moreover it is able to analyze up to eight samples simultaneously by different iTRAQ labels [11]. Besides, 2D–LC–MS/MS is high-resolution that allow measure proteins at low levels with high precision and specificity [12, 13]. In the present study, we took advantages of those technologies to demonstrate the effect of high-fat diet and metformin on lipid metabolism, and to further identify potential key proteins involved in energy metabolism with metformin intervention.

## Materials and methods

### Animals

All animals were handled according to the Standards for Laboratory Animals (GB14925-2001) and the Guideline on the Humane Treatment of Laboratory Animals (MOST 2006a) established by the People’s Republic of China. The two guidelines were conducted in adherence to the regulations of Institutional Animal Care and Use Committee (IACUC) and all animal procedures were approved by Beijing Administration Office of Laboratory Animal (approval number: SCXK-Beijing- 2009-0004). All efforts were made to minimize suffering. Six- to eight-week-old female C57BL/6J mice were purchased from HFK Bioscience Laboratories (Beijing, China). All mice were maintained under SPF conditions and 12 h light/dark cycle at 23 ± 2 °C, with free access to water and diet. Mice were divided into two groups: one was fed with standard chow (SC; 10% lipids) diet and another group was fed with high-fat diet (HFD; 45% lipids) for 22 weeks. Food intake for 24 h and body weight (once per week) were dynamically monitored. After 22 weeks, body weight of mice in HFD group was increased by 65.7% when compared to normal diet (ND) mice. Then obese mice fed HFD were randomized into two groups, one was control group (HFD,ddH2O 200 mg/kg BW, qd, i.g.), the other was treated with metformin (MET, 200 mg/kg BW, qd, i.g.) for 8 weeks.

### Oral glucose tolerance test (OGTT) and hyperinsulinemic–euglycemic clamp

For OGTT, 5 h fasted mice were given oral glucose (2.0 g/kg body weight, I.G.). Blood was taken at 0 min and 30, 60 and 120 min after glucose was given. Blood glucose concentrations were measured by glucose oxidase method [14]. Plasma levels of total cholesterol (TC) and triglyceride (TG) were tested by enzymic method (Biosino bio-technology & science Inc, China). Hyperinsulinemic–euglycemic clamp were performed after 8 weeks of metformin therapy. With overnight fasted, mice were anesthetized with 80 mg/kg pentobarbital sodium, and inserted a single implantation tubing into the right jugular vein for infusion of insulin or glucose. Indwelling catheter infused with constant rate of human insulin (20 mIU/kg/min) and variable rate of glucose solution (25%, w/v) to maintain blood glucose level at 5.5 ± 0.5 mmol/L. The insulin sensitivity was measured by glucose infusion rate (GIR) during the last 80 min [15].

### Preparation of BAT and WAT samples

Inter scapular BAT and gonadal WAT of mice among three groups were excised and washed with a cold saline solution. BAT and WAT was snap frozen in liquid nitrogen and kept at − 80 °C until analysis. Total WAT and BAT proteins, extracted in each group, were pooled at the same amount respectively.

### iTRAQ labeling

BAT and WAT peptide samples were respectively labeled with iTRAQ reagent as follows: The digested ND samples were considered as internal standard. The internal standard, HFD and MET samples were labeled by 114, 115, 116 iTRAQ. Labeling was performed according to the manufacturer’s protocol (ABsciex, Massachusetts, USA) [16]. The ND, HFD and MET samples were mixed into one sample at the same amount and lyophilized.

### LC–MS/MS

The pooled mixture from labeled samples was first fractioned by high-pH RPLC column from Waters (4.6 mm × 250 mm, C18, 3 μm). Triple TOF 5600 were used to analyze the sample. The MS data were acquired with high sensitivity mode using the following parameters: 30 data-dependent MS/MS scans per every full scan; full scans was acquired at resolution 40,000 and MS/MS scans at 20,000; 35% normalized collision energy, charge state screening (including precursors with +2 to +4 charge state) and dynamic exclusion (exclusion duration 15 s); MS/MS scan range was 100–1800 m/z and scan time was 100 ms.

### Database search

The MS/MS spectra were respectively searched against the SwissProt mouse database from Uniprot website (http://www.uniprot.org) using Mascot software version 2.3.02 (Matrix Science, UK). Scaffold was used to further filter the database search results by decoy database method. The following filter was used in this study, 1% false positive rate at protein level and each protein with 2 unique peptides. After filtering the results by above filter, the peptide abundances in different reporter ion channels of MS/MS scan were normalized.

### Gene ontology (GO) functional analysis

All differential proteins identified by two approaches were assigned their gene symbol via the PANTHER database (Protein Analysis through Evolutionary Relationships, http://www.pantherdb.org/). Protein classification was performed based on their functional annotations using GO for cellular component, biological process, and molecular function. When more than one assignment was available, all of the functional annotations were considered in the results.

### IPA network analysis

All differential proteins were used for pathway analysis. For this purpose, the SwissProt accession numbers were inserted into the Ingenuity Pathway Analysis (IPA) software (Ingenuity Systems, Mountain View, CA). This software categorizes gene products based on the location of the protein within cellular components and suggests possible biochemical, biological and molecular functions. Furthermore, proteins were mapped to genetic networks available in the Ingenuity and other databases and ranked by score. These genetic networks describe functional relationships between gene products based on known interactions in literature. Through the IPA software, the newly formed networks were associated with known biological pathways.

### Western blotting

BAT or WAT protein was extracted with a detergent (Applygen technology Inc, China) and lysed in buffer solution containing 7 M urea, 2 M thiourea, 65 mM DTE, 83 mM Tris (Sigma-Aldrich, St. Louis, MO, USA). Protein content of adipose tissues was determined by the Bradford method with Bradford reagents (Thermo Fischer Scientific, USA). Protein solution and loading buffer were mixed in proportion followed the instructions. The antibodies were used as follows: anti-rabbit CPT2 (1:1000), AIF1 (1:500), RAS (1:200), CPT1a (1:1000), CPT1b (1:500), anti-mouse β-actin (1:4000; Sigma–Aldrich, USA). After washing with TBST, the membranes were incubated with HRP conjugated goat anti-rabbit IgG (1:5000; Earthox LLC., San Francisco, California, USA) and anti-mouse IgG secondary antibody (1:3000; Cell Signaling Technology, Inc., Danvers, USA). Western blot was analyzed by scanning with LAS4000 (Fujifilm, Tokyo, Japan) and the data were analyzed using Image J software. β-actin was used as a normalization control. Duplicate experiments were carried out for all proteins.

### Data analysis and statistics

Data derived from six mice in ND, HFD and MET group were presented as mean ± SEM. Data distribution was assessed by qq-plots and homogeneity of variance was checked before one-way ANOVA. Statistical analysis was performed using SPSS 17.0 software. All differential proteins were analyzed by the Ingenuity Pathway Analysis. Differences with p values < 0.05 were considered to be statistically significant.

## Results

**Fig. 1:**
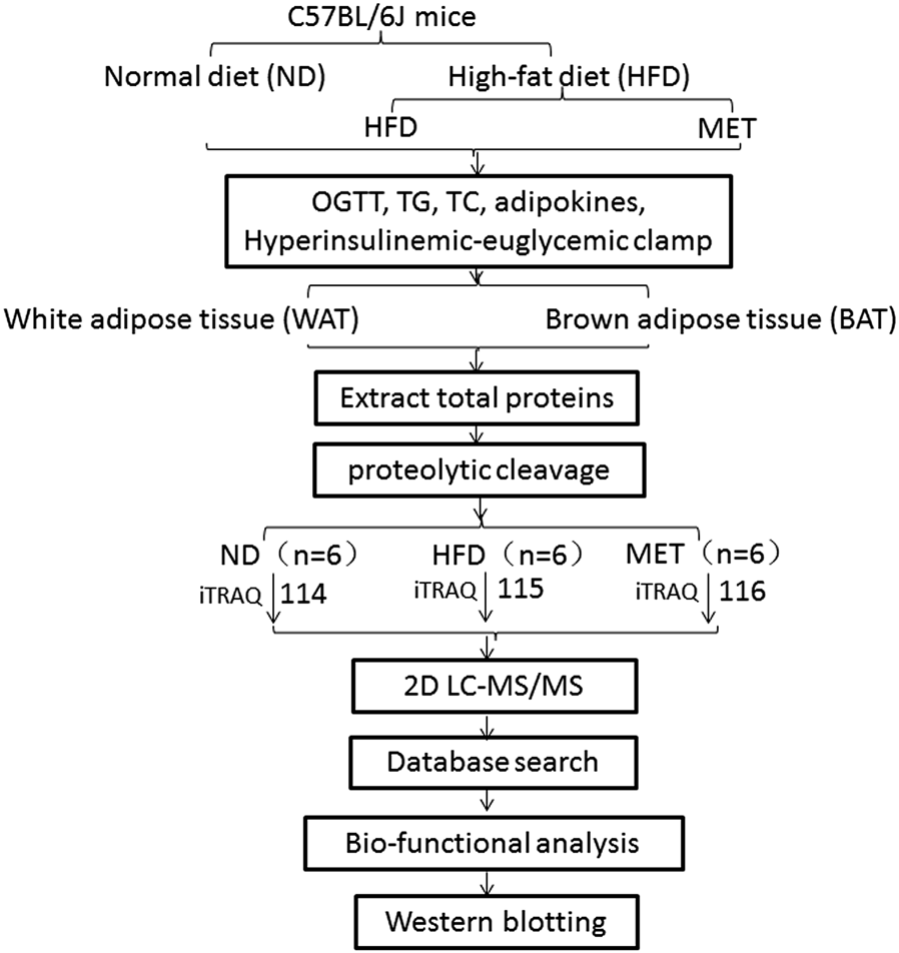
Work flow of the present study. C57BL/6J mice were fed with normal diet (ND) or high-fat diet (HFD) for 22 weeks. Six of HFD-induced obese mice were treated with metformin. After treatment for 8 weeks, oral glucose tolerance test (OGTT) and hyperinsulinemic–euglycemic clamp were performed to evaluate the improvement of glucose tolerance and insulin resistance. Protein expressions of WAT and BAT in mice among ND, HFD, and MET group were identified and quantified with iTRAQ-coupled 2D LC-MS/MS. The results were analyzed by MASCOT, Scaffold and IPA. Finally, western blot analysis was performed to validate the variation of the key differential proteins

In the study, C57BL/6J mice were induced to obesity by feeding HFD for 22 weeks. Then metformin was administered to obese mice for 8 weeks, moreover, mice fed normal diet were considered as control group. Body weight and food intake of mice were measured per week. Oral glucose tolerance test (OGTT) and hyperinsulinemic–euglycemic clamp were performed to evaluate glucose metabolism and insulin sensitivity of mice. Blood lipids and serum level of adipokines were tested to assess lipid metabolism and inflammation state in different groups of mice. Using iTRAQ-coupled 2D LC–MS/MS, with normal diet group as a control, we compared and functional analyzed the differential expressed proteins in WAT and BAT among HFD and MET group. In addition, western blotting was applied to validate several potential key proteins (Fig. 1).

### Metformin improved glucose tolerance and insulin sensitivity

As compared with normal group (25.2 ± 1.0 g), body weight of mice fed HFD (41.1 ± 1.6 g) was increased 63%. Metformin inhibited food intake (Additional file 1: Figure S1) and induced weight loss. The body weight of obese mice was reduced by 10.9% with metformin (Additional file 1: Figure S2).

In order to evaluate effects of metformin on glucose tolerance, OGTT was performed on these three groups of mice. The area under curve (AUC) of blood glucose in HFD group was larger than which in ND group, while AUC in MET group was decreased significantly, as compared with HFD group (Additional file 1: Figure S3).

Hyperinsulinemic–euglycemic clamp was applied to assess insulin sensitivity, which was represented by GIR. The GIR of HFD group was lower than that of ND group. The results were similar to our previous data [4]. The GIR in MET group were increased significantly compared with HFD group (Additional file 1: Figure S4).

### Metformin benefited for blood lipids and adipokines

The level of TC and TG in metformin group was lower than HFD group. In obese mice, the serum leptin was increased dramatically and the level of adiponectin in serum was reduced significantly. In addition, metformin lowered the level of leptin and raised the concentration of adiponectin significantly. Resistin was not changed significantly among different groups. Resistin was also identified in WAT; in addition, adiponectin was identified in both WAT and BAT by LC–MS/MS. The variation tendency of both adipokines in adipose tissue was in accord with the serum level tested through ELISA. However, leptin was only detected by ELISA, but identified neither in WAT nor in BAT by LC–MS/MS that might due to the limitation of methodology (Table 1).

**Table 1:** Body weight, adipose tissue weight and metabolic profile of C57BL/6J mice among different groups (mean ± SEM)

	ND	HFD	MET
WAT mass (%)	2.16 ± 0.38**	4.74 ± 0.57	4.56 ± 0.34
BAT mass (%)	0.26 ± 0.04*	0.14 ± 0.02	0.20 ± 0.03
TC (mg/dl)	38.95 ± 0.82***	76.20 ± 3.04	72.71 ± 1.88
TG (mg/dl)	61.45 ± 1.70**	75.88 ± 3.15	62.38 ± 1.39**
Leptin (pg/ml)	687.56 ± 50.46***	1144.19 ± 70.59	707.91 ± 48.81***
Adiponectin (ng/ml)	4.98 ± 0.13***	3.34 ± 0.26	4.09 ± 0.15*
Resistin (pg/ml)	793.91 ± 84.14	811.32 ± 75.58	667.40 ± 47.91

*ND* normal diet, *HFD* high fat diet, *MET* metformin, *WAT* white adipose tissue, *BAT* brown adipose tissue, *TC* total cholesterol, *TG* triglyceride. *Compared with HFD group.*p < 0.05, **p < 0.01, ***p < 0.001

### Protein expression profiling of WAT and BAT

To identify how metformin altered proteome of adipose tissue in obese mice, we applied iTRAQ-coupled with 2D LC–MS/MS. Each protein was determined by Mascot search against the Swissprot mouse database, and the iTRAQ quantitative analysis was performed by scaffold. 3469 and 2734 proteins were quantified in WAT and BAT, respectively. The criteria were followed to determine the proteins: two or more high confidence unique peptides had to be identified; false positive rate of the identification of protein or peptide was less than 1%. To diminish technical error, proteins with coefficient of variation (CV) for two runs > 0.25 were excluded (Additional file 1: Figure S5, S6). With ND group as a control, a fold change of ≥ 1.5 was assigned for the iTRAQ ratio threshold to minimize biological and technical errors (Additional file 2: File S1 Dataset; Additional file 3: File S2 Dataset).

### Global functional annotations of the quantified proteins in WAT and BAT

**Fig. 2:**
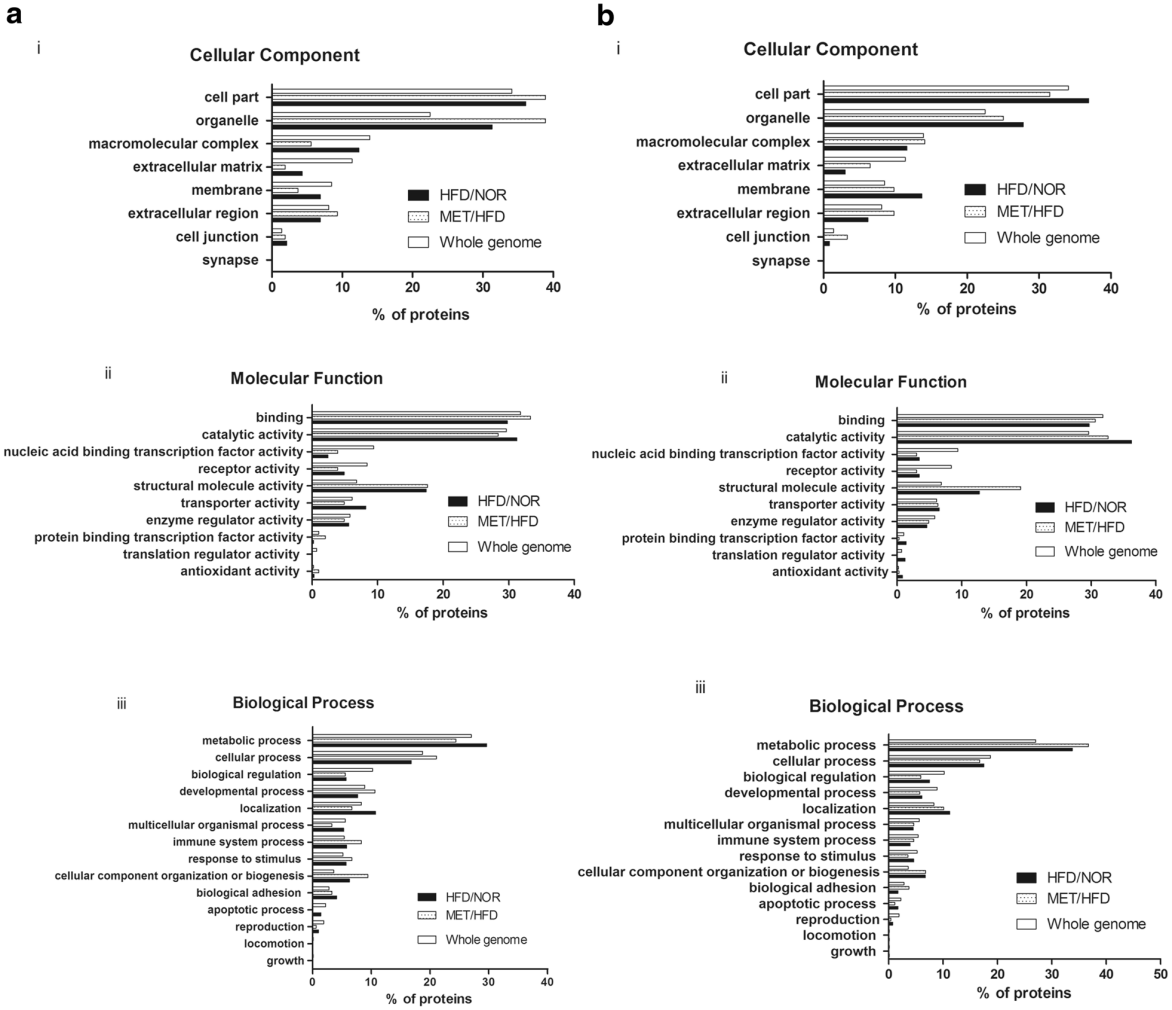
We compared differential proteins from **a** WAT and **b** BAT between HFD and MET group through the PANTHER classification system. (i) Cell Component of whole genome and the differentially expressed proteins between ND and HFD, HFD and MET group. (ii) Molecular Function assigned to whole genome and the differentially expressed proteins between ND and HFD, HFD and MET group. (iii) Biological processes assigned to whole genome and the differentially expressed proteins between ND and HFD, HFD and MET group

Using GO database, differential proteins were categorized by cell component, molecular function and biological process. 36.1% WAT proteins and 36.9% BAT proteins in HFD group belonged to cell part. For WAT, the proportions of cell part were increased in MET group, while they were decreased in BAT. 31.2% WAT proteins and 36.2% BAT proteins in HFD group were annotated as catalytic activity. Metformin decrease the percentage of proteins that assigned to catalytic activity in WAT and BAT. 29.6% and 24.4% WAT proteins in HFD and MET group were involved in metabolic process. 33.8% and 36.7% BAT proteins in HFD and MET group were related to metabolic process. As compare with whole genome profile, it revealed that more proteins in BAT were took part in metabolism (Fig. 2a, b).

In addition, functions of differential proteins were further analyzed by Ingenuity Pathway Analysis. With ND group as a control, we compared HFD and MET group. The related disease & biological function were ranked according to the degree of correlation. In WAT, the top five related functions were lipid metabolism, molecular transport, small molecule biochemistry, cardiovascular disease and carbohydrate metabolism. In BAT, they were lipid metabolism, molecular transport, small molecule biochemistry, cancer, and development disorder.

The statistically significant pathways were also analyzed. Differentially expressed proteins in WAT were predominantly in pathway of EIF2 signaling, LXR/RXR activation and actin cytoskeleton signaling. In the pathway of EIF2 signaling (-log (p value) was 17.5), Phosphatidylinositol 3-kinase catalytic subunit type 3 (Pik3c3) as well as 40 s and 60 s ribosomal subunit were 1.6 fold up-regulated in HFD group, and were 1.4 fold up-regulated in MET group. Moreover, the pathway of EIF2 signaling in BAT was diverse since cluster of GTPase HRas (Hras1) was 1.5 fold up-regulated in HFD group, but metformin intervention decreased Hras1 to normal level.

In contrast, proteins in BAT were characterized by the pathway of mitochondrial dysfunction and oxidative phosphorylation. In the pathway of mitochondrial dysfunction (−log (p value) was 28), apoptosis-inducing factor 1 (AIF1) was 2.3 fold up-regulated in HFD group, while it was 1.65 fold up-regulated in MET group. Voltage-dependent anion-selective channel protein 1–3 (Vdac1–3), carnitine O-palmitoyltransferase1b (CPT1b), CPT2 and dihydroorotate dehydrogenase (quinone), mitochondrial (Dhodh) were up regulated, whereas protein DJ-1 (Park7), peroxiredoxin-1, 2, 3 (PRDX) and caspase-3 were down regulated. In addition, differential expressed proteins involved in mitochondrial dysfunction of WAT mainly including, the up-regulation of carnitine O-palmitoyltransferase1a (CPT1a), CPT1b, Cytochrome b5 (CYB5b), and the down-regulation of Cluster of Alpha-synuclein (Snca) and Pyruvate dehydrogenase E1 component subunit alpha, somatic form, mitochondrial (Pdha1).

### Confirmation of potential key proteins by western blotting

**Fig. 3:**
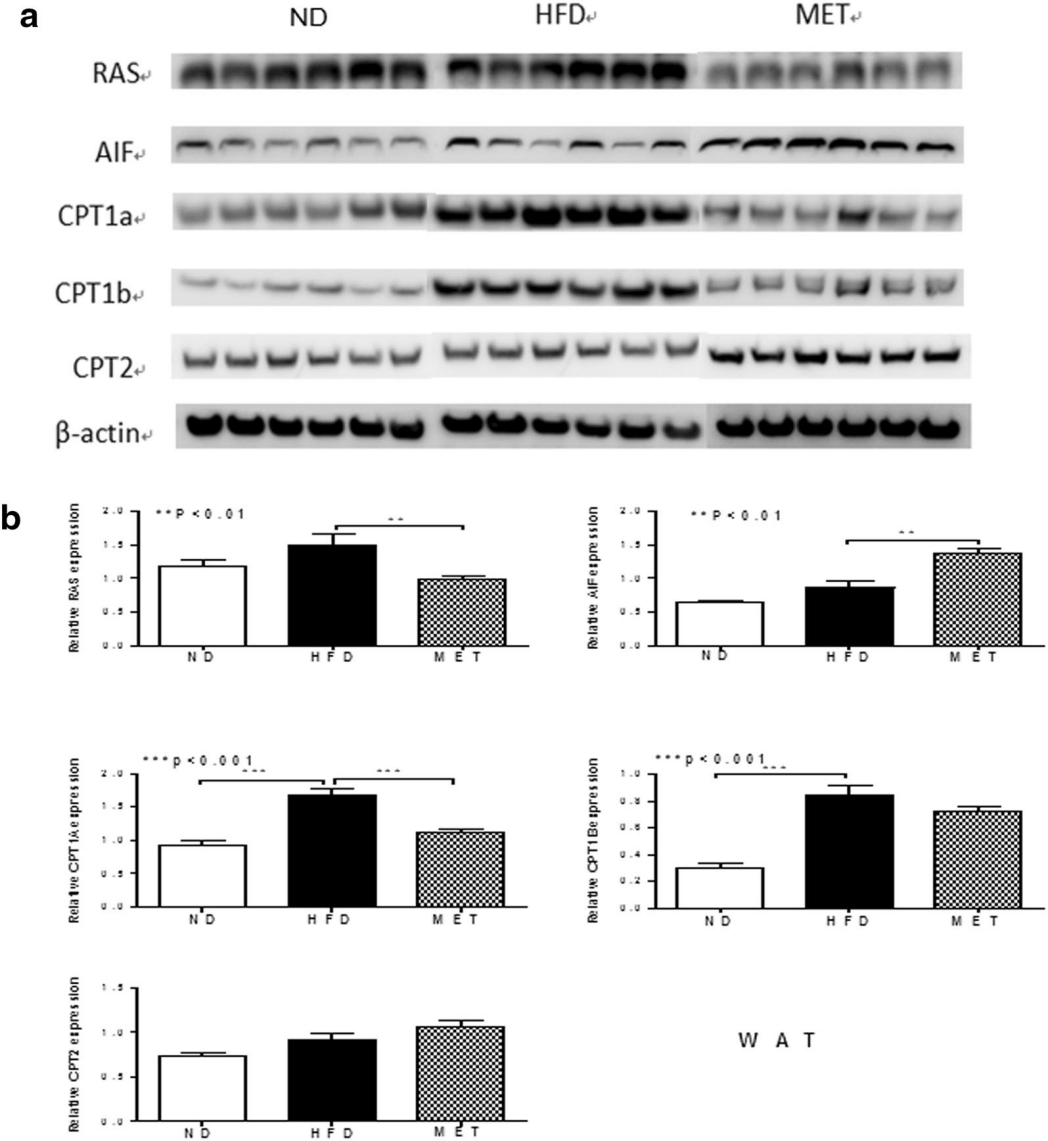
Western blotting was performed to validate proteomic data for some WAT proteins of interest and differential expressed in ND, HFD and MET groups. **a** Gel images of western blotting in ND, HFD and MET groups (n = 6/group). **b** β-actin was used as a normalization control. Compared normalized density values from blots among ND, HFD and MET groups. HFD group was considered as the control group. RAS, cluster of GTPase HRas; AIF, apoptosis-inducing factor 1; CPT1a, carnitine O-palmitoyltransferase1a; CPT1b, carnitine O-palmitoyltransferase1b; CPT2, carnitine O-palmitoyltransferase 2

Through functional analysis of differential proteins in WAT and BAT among different groups, and also based on previous data, five proteins involved in mitochondrial dysfunction and EIF2 signaling were verified by western blotting. In WAT, although HFD did not alter Hras1 obviously, metformin down regulated it statistically significant. AIF1 was up-regulated in MET group. HFD up regulated CPT1a and CPT1b significantly, while metformin down regulated the expression of CPT1a. CPT2 was not changed among different groups in WAT (Fig. 3a, b).

**Fig. 4:**
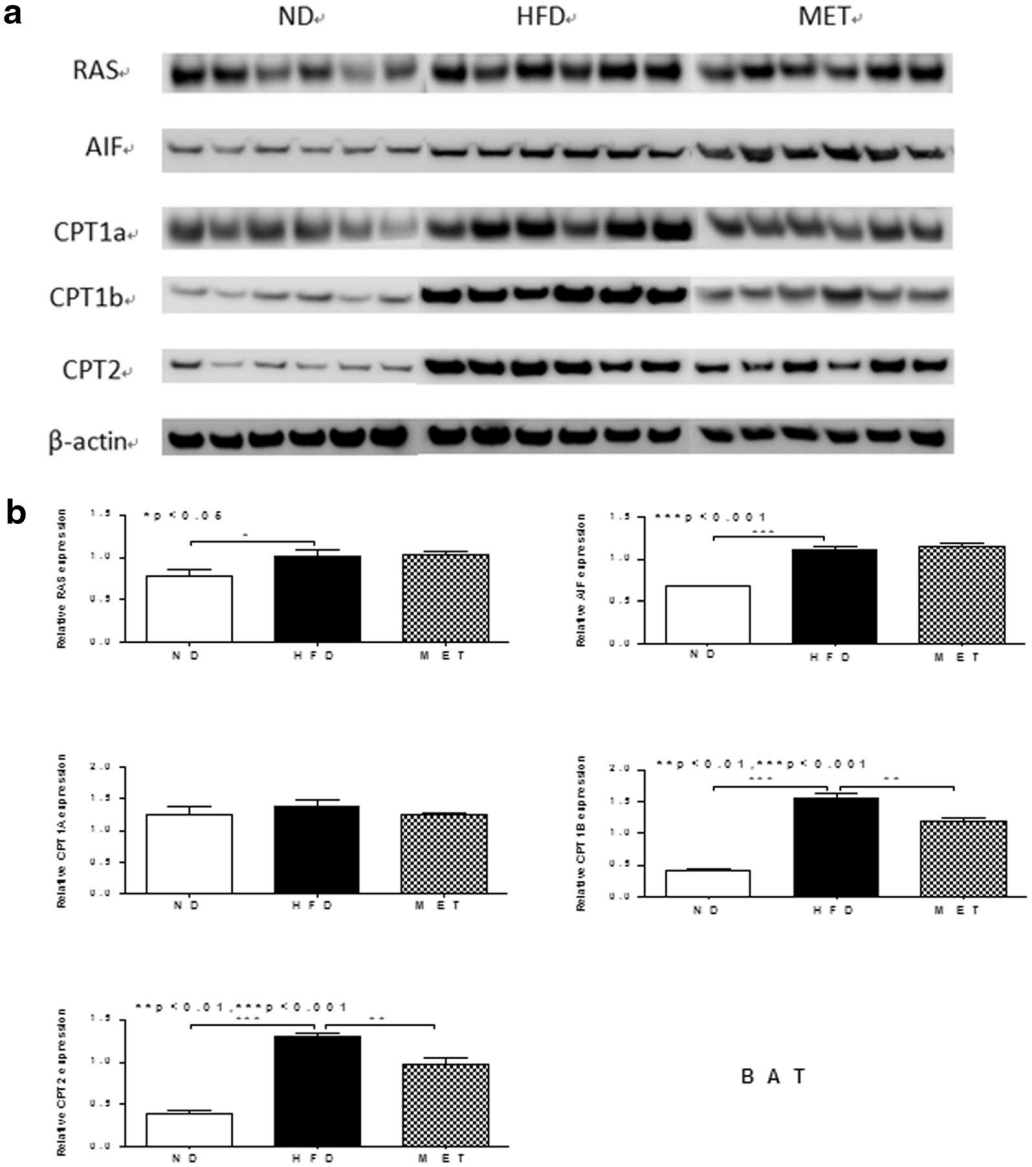
Western blotting was performed to validate proteomic data for some BAT proteins of interest and differential expressed in ND, HFD and MET groups. **a** Gel images of western blotting in ND, HFD and MET groups (n = 6/group). **b** β-actin was used as a normalization control. Compared normalized density values from blots among ND, HFD and MET groups. HFD group was considered as the control group

As for BAT, HFD up regulated Hras1 and AIF1, and those were not affected by metformin. CPT1b and CPT2 were up-regulated by HFD, while they were down-regulated by metformin significantly. However, CPT1a did not alter by different interventions (Fig. 4a, b).

## Discussion

Metformin is widely used for diabetes in the world. It was found that metformin is effective for the control of body weight and improvement of insulin sensitivity [17]. However, the effect of metformin on adipose tissue in obesity is less clear. In the current study, we explored the potential targets of metformin on energy metabolism.

### Metformin improved insulin sensitivity and regulated adipokines

It was reported that high-dose metformin increased insulin sensitivity in high-fat fed rats [18]. In our study, obese mice were treated with metformin for an appropriate dose. We found that the body weight of mice was reduced obviously by metformin, and hyperinsulinemic–euglycemic clamp showed that metformin improved insulin sensitivity. There were researches demonstrated that metformin ameliorated insulin sensitivity in rodents by activating AMP-activated protein kinase [4]. Consequently, it might explain that metformin is benefit for insulin sensitivity in human. However, further researches are required to determine whether the improvement of insulin sensitivity by metformin is independent from weight loss.

Leptin, functions as a pro-inflammatory adipokine, regulates appetite through the central nervous system and the serum leptin positively correlates with adipose mass [19]. Metformin may ameliorate chronic inflammation by decreasing the level of leptin. Besides, adipocytes synthesize and secrete anti-inflammatory adipokines like adiponectin, which was decreased in obesity. A positive correlation was found between plasma adiponectin and insulin sensitivity [20]. Here the variation tendency of adiponectin in different group, indicating the insulin resistance was increased by HFD but improved by metformin. Resistin is a pro-inflammatory adipokine, in addition it regulates insulin resistance and inflammation via IL-6 and TNF secretion from macrophages [21, 22]. Resistin in our study was decreased in obese mice, indicated that insulin resistance may not evoked by resistin.

### Metformin ameliorated fatty acid metabolism in WAT and BAT

Based on our previous study, HFD up regulated CPT1b and CPT2 in BAT, and it indicated that HFD might increase the compensated energy expenditure in BAT [23]. CPT1 regulates mitochondrial beta-oxidation, and this protein is highly expressed in liver, muscle, adipose tissue and brain [24]. CPT1a is liver isoform, while CPT1b is muscle isoform [25]. Some studies indicated that the different expressed of CPT1 is depended on species, gender or tissue [26–28]. In the present study, we found that HFD stimulated fatty acid β-oxidation through up-regulated CPT1a and CPT1b in WAT. Interestingly, with metformin intervention, CPT1a was down regulated in WAT significantly, while CPT1b was not affected obviously. As for BAT, we discovered CPT1b and CPT2 were both down regulated by metformin. Those implied that metformin might improve energy metabolism in WAT and BAT,then attenuate the compensated energy expenditure. Furthermore, CPT1a might be predominant expressed in WAT, while CPT1b and CPT2 were more important with fatty acid metabolism in BAT.

### Metformin may improve endoplasmic reticulum stress in WAT

Among differentially expressed proteins, according to IPA, the highest-scoring pathway in WAT was EIF2 signaling. EIF2 signaling is associated with endoplasmic reticulum stress [29], which is one of pathomechanisms of metabolic diseases, including type 2 diabetes mellitus, hypertension, dyslipidemia and coronary artery disease [30]. In the present study, we found that Pik3c3, a catalytic subunit of the phosphatidylinositol 3-kinases (PI3 Ks), was increased in WAT of mice fed HFD as compared to ND. PI3Ks consist of three classes of isoforms. Pik3c3, the only class III PI3K, was also known as vacuolar protein sorting 34 (VPS34), which was first identified in saccharomyces cerevisiae as the gene product involved in the trafficking vesicles from the Golgi complex towards the vacuole [31]. It has been shown that Pik3c3 is a nutrient-regulated lipid kinase that regulates glucose and amino acid for protein synthesis and cell growth through mTOR-S6K1 signaling pathway [32, 33]. In addition, there was evidence that Pik3c3 regulated autophagy activity and correlated with endoplasmic reticulum stress [34]. Here in the study, high-fat diet increased Pik3c3 and 40 s/60 s ribosomal subunit downstream. This is an indicator that overload of lipid may stimulate cell proliferation and energy storage by activating Pik3c3 in white adipocytes. The expressions of Pik3c3 in WAT were normalized via treated with metformin, which suggested that metformin may improve endoplasmic reticulum stress and be benefit for lipid metabolism.

## Conclusions

In summary, we discovered that metformin might affect energy metabolism in adipose tissue, and improve endoplasmic reticulum stress in WAT. Besides, metformin improved fatty acid metabolism via CPT in WAT and BAT. CPT1a might be an important enzyme for WAT, while CPT1b and CPT2 were more predominant for BAT in lipid metabolism.

## Supplementary information

**Additional file 1: Figure S1.** Average food intake per day among ND, HFD and MET groups was calculated. Metformin decreased food intake of mice. **Figure S2** Weight changes of mice among ND, HFD and MET groups were measured per week. HFD group was considered as the control group. **Figure S3** OGTT. 5 h fasted mice from ND, HFD and MET groups were given glucose (2.0 g/kg body weight, I.G.) for an OGTT. Blood was taken at 0 min and 30, 60 and 120 min after glucose was given. Blood glucose concentrations were measured by glucose oxidase method. Results are given as mean ± SEM. HFD group was considered as the control group. **Figure S4** With overnight fasted, hyperinsulinemic–euglycemic clamp was conducted and lasted for 120 min. The insulin sensitivity was measured by glucose infusion rate (GIR) during the last 80 min. The glucose infusion rate in MET group was increased significantly compared with HFD group. **Figure S5** Distribution of the coefficient of variation (CV value) in WAT from two run assays in LC-MS/MS was exhibited. In order to minish technical error, proteins whose CV value greater than 0.2 were excluded. **Figure S6** Distribution of the coefficient of variation (CV value) in BAT from two run assays in LC-MS/MS was exhibited. In order to minish technical error, proteins whose CV value greater than 0.2 were excluded.

**Additional file 2: File S1 Dataset.** Scaffold report for proteins in WAT identified by iTRAQ coupled with 2D LC–MS/MS.

**Additional file 3: File S2 Dataset.** Scaffold report for proteins in BAT identified by iTRAQ coupled with 2D LC–MS/MS.

## Data Availability

All data generated or analysed during this study are included in this published article.

## Abbreviations

WAT: white adipose tissue
BAT: brown adipose tissue
ND: normal diet
HFD: high-fat diet
OGTT: oral glucose tolerance test
UCP-1: uncoupling protein 1
NPY: neuropeptide-Y
FGF21: fibroblast growth factor 21
GLP-1: glucagon-like peptide-1
AIF1: apoptosis-inducing factor 1
CPT: carnitine O-palmitoyl transferase
iTRAQ: isobaric tag for relative and absolute quantification
LC–MS/MS: liquid chromatography-tandem mass spectrometry
TC: total cholesterol
TG: triglyceride
GIR: glucose infusion rate
GO: gene ontology
IPA: ingenuity pathway analysis
AUC: area under curve
CV: coefficient of variation
Pabpc1: polyadenylate-binding protein 1
Vdac: voltage-dependent anion-selective channel protein
