# Supplementary figures and images for: Effects of metformin on metabolism of white and brown adipose tissue in obese C57BL/6J mice
Source: Diabetol Metab Syndr. 2019 Nov 27;11:96. doi: 10.1186/s13098-019-0490-2 (PMC6880501; doi:10.1186/s13098-019-0490-2)

## Slide 1
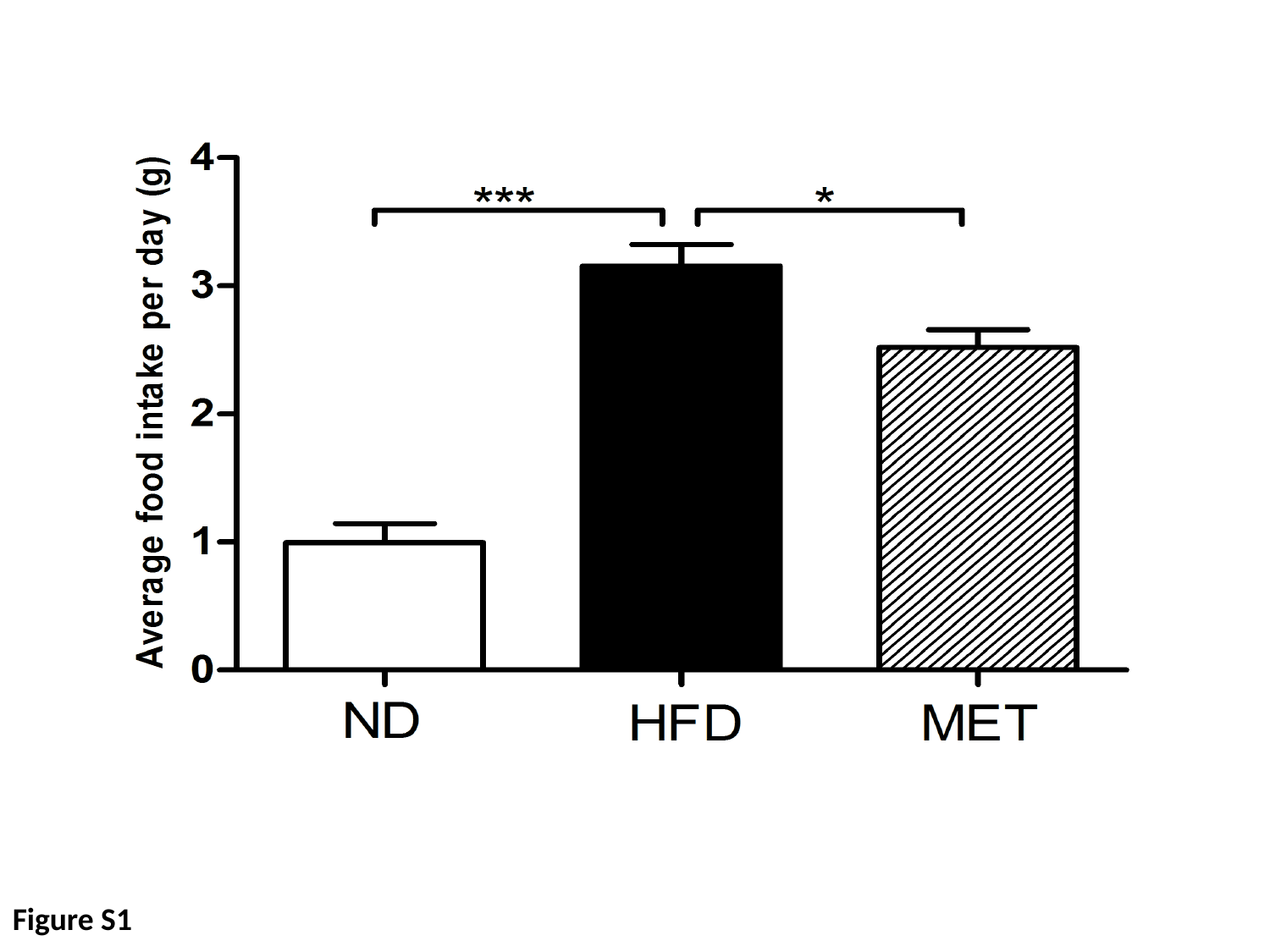

Figure S1

## Slide 2
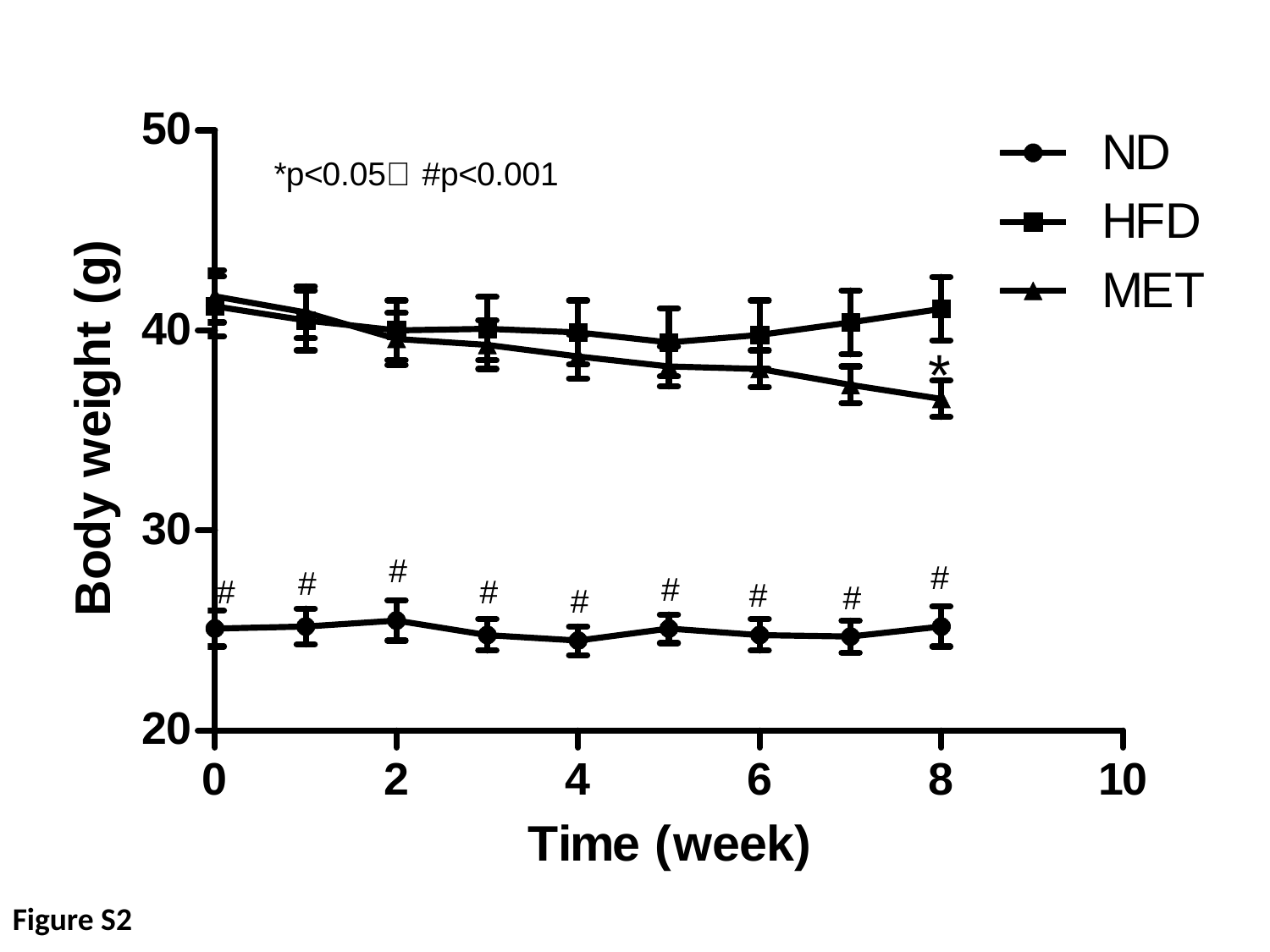

Figure S2

## Slide 3
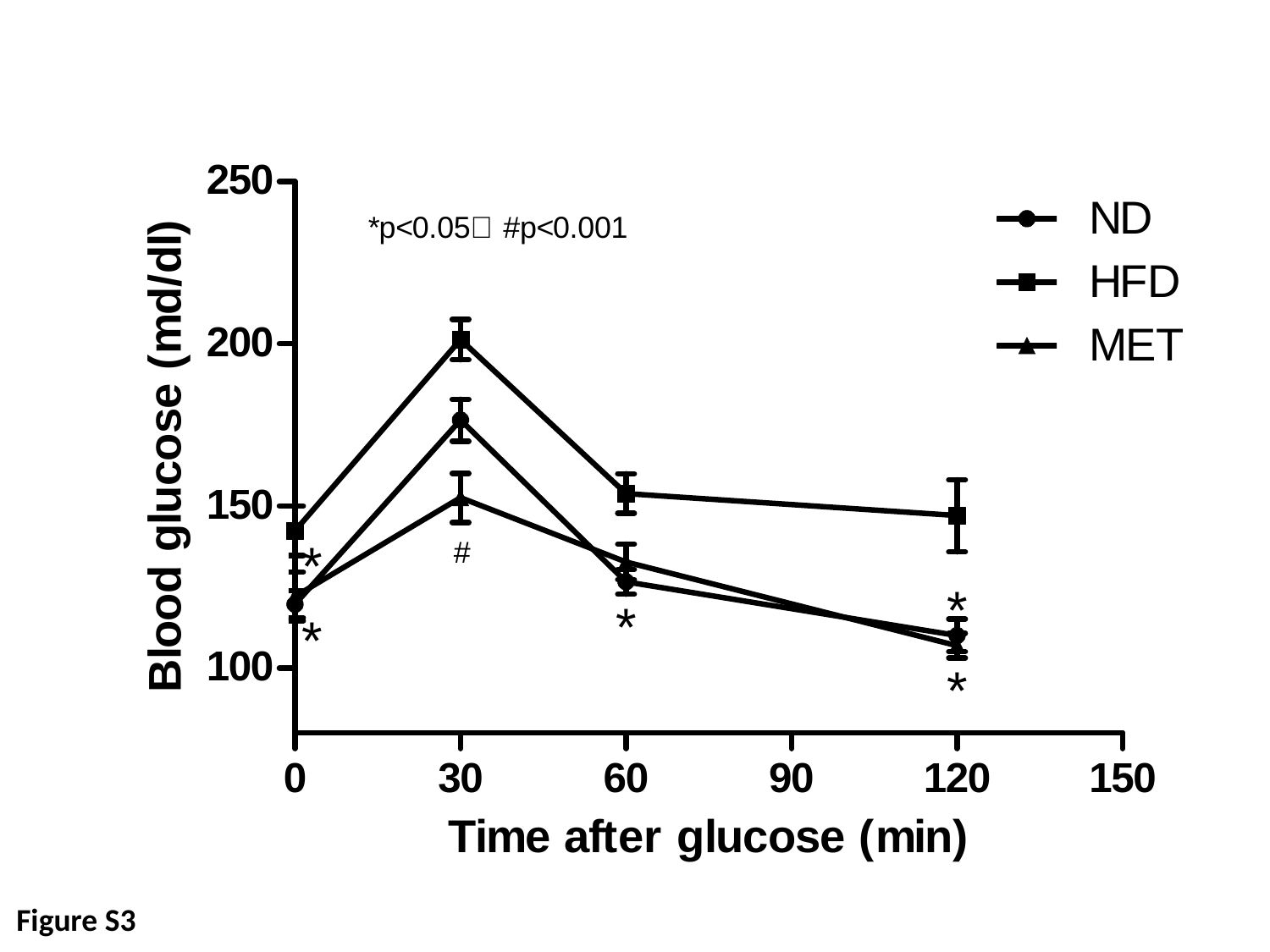

Figure S3

## Slide 4
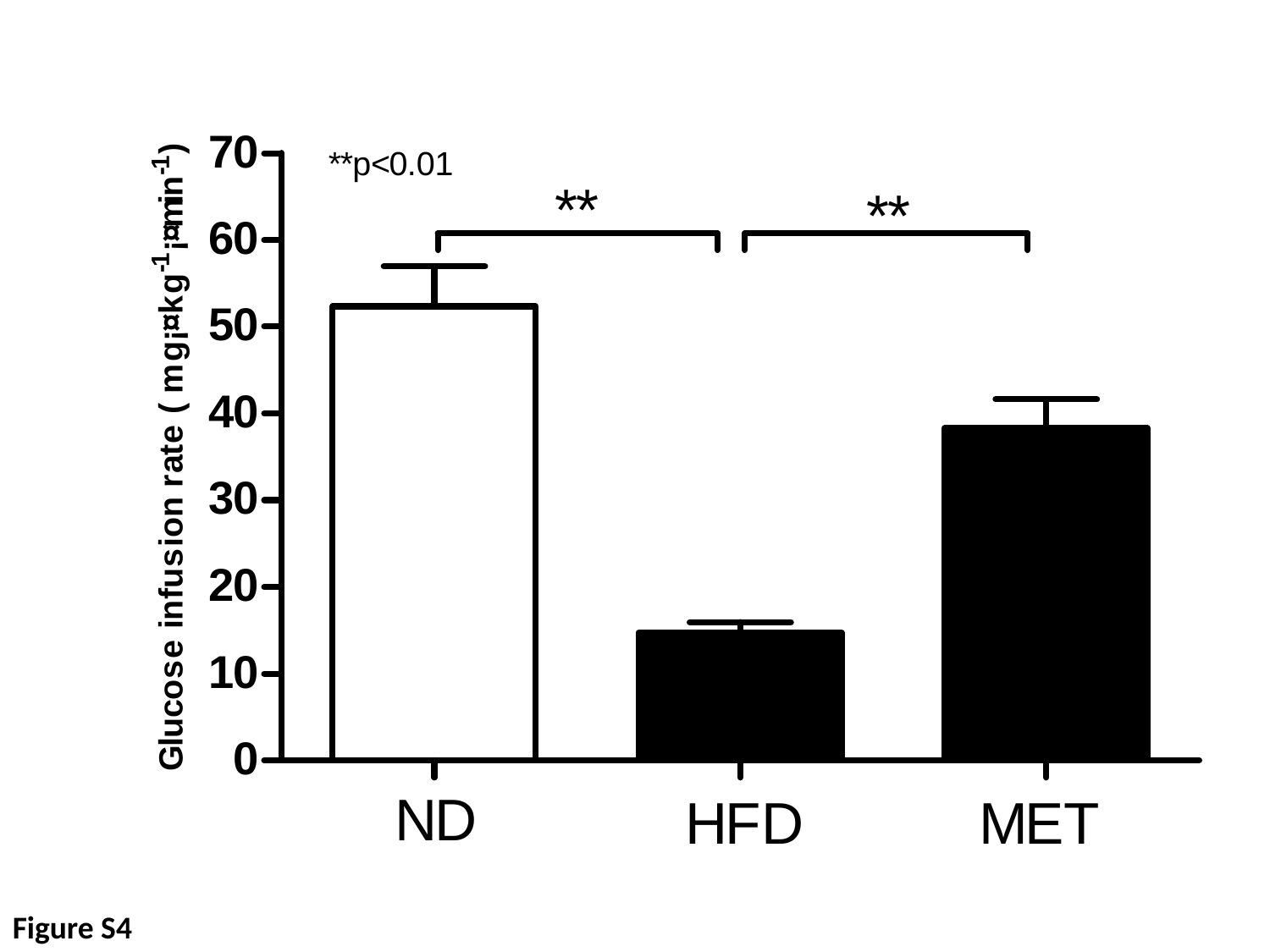

Figure S4

## Slide 5
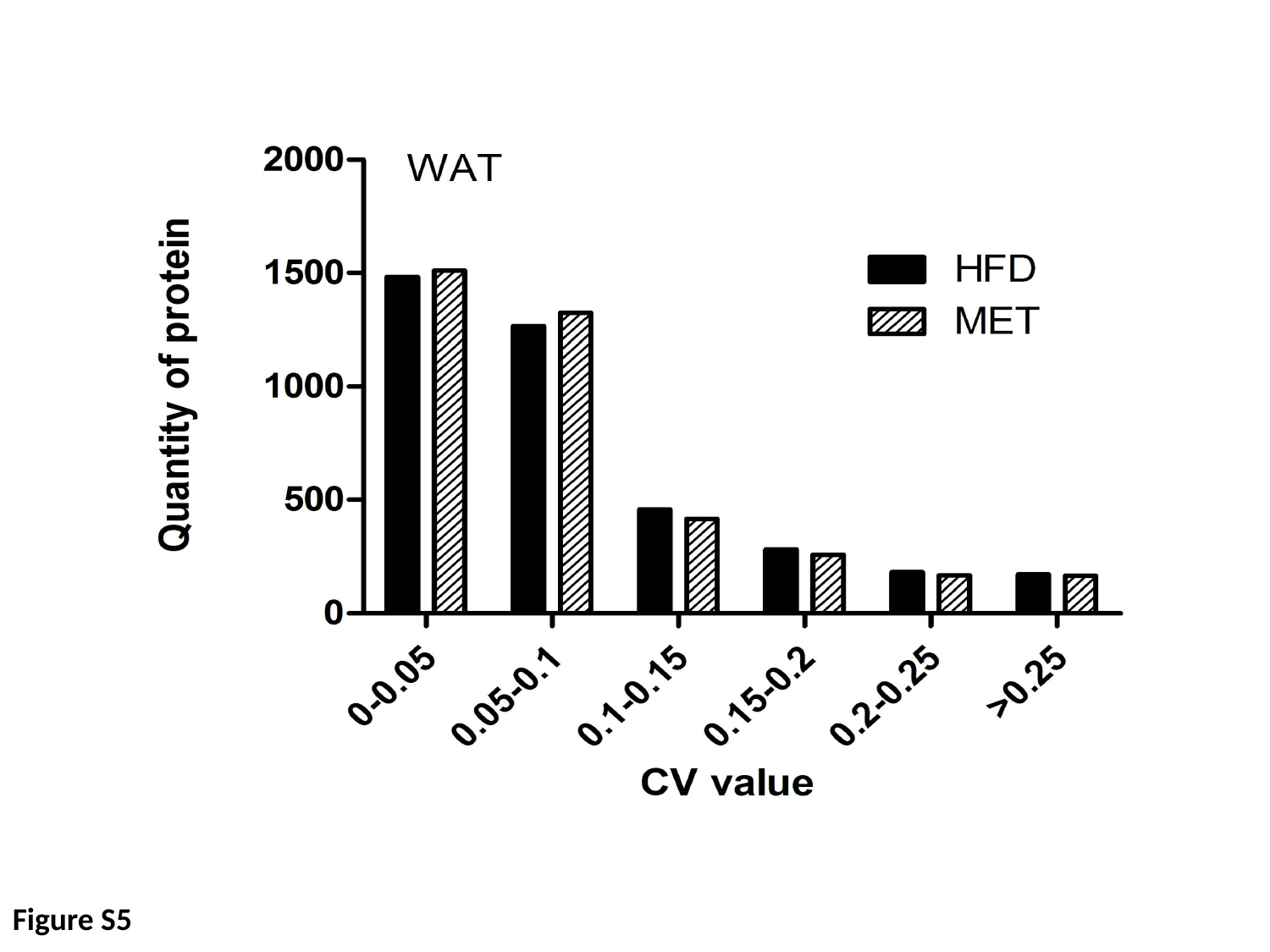

Figure S5

## Slide 6
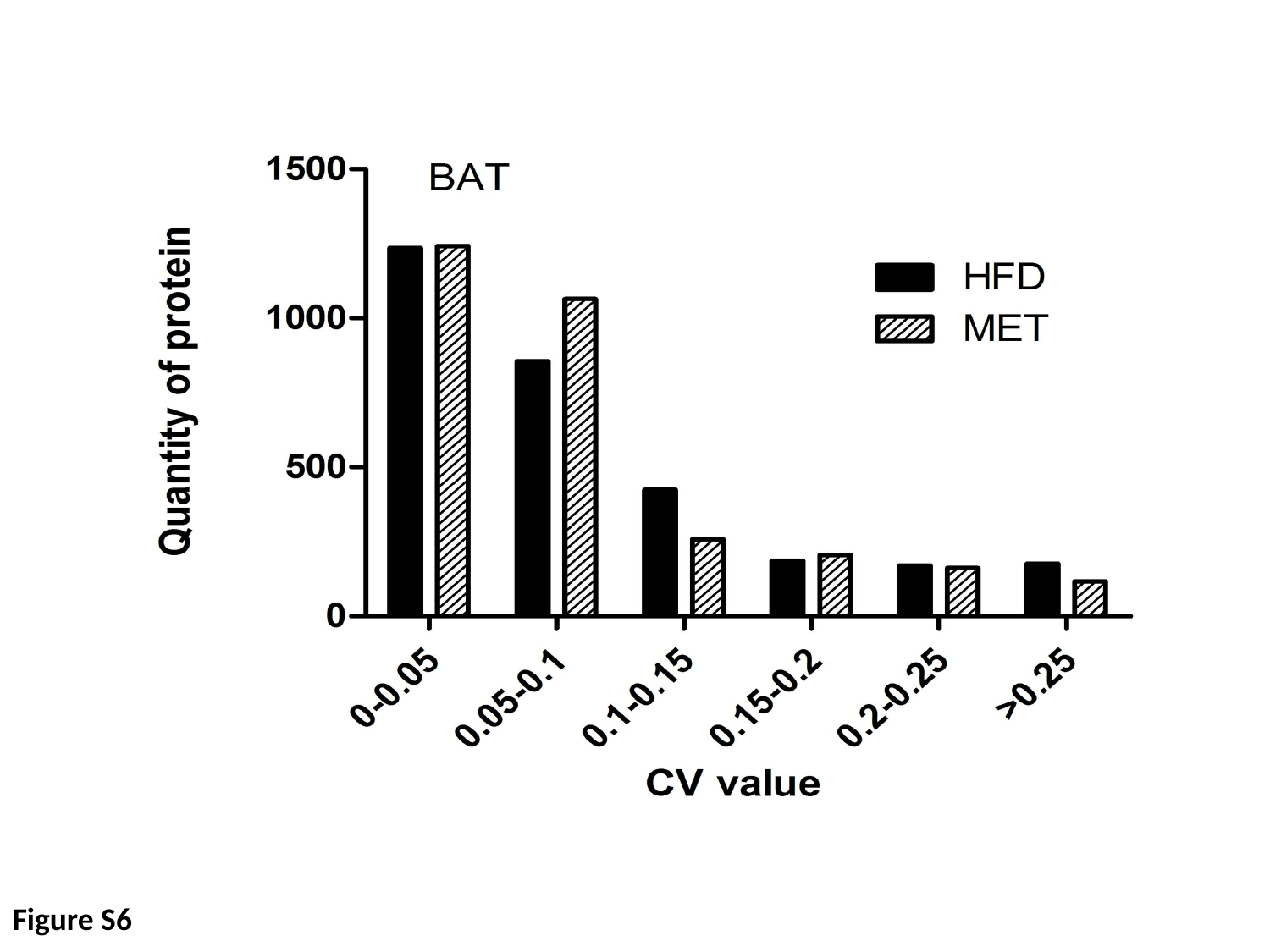

Figure S6

Supplement: Supplementary file 1 — Additional file 1: Figure S1. Average food intake per day among ND, HFD and MET groups was calculated. Metformin decreased food intake of mice. Figure S2 Weight changes of mice among ND, HFD and MET groups were measured per week. HFD group was considered as the control group. Figure S3 OGTT. 5 h fasted mice from ND, HFD and MET groups were given glucose (2.0 g/kg body weight, I.G.) for an OGTT. Blood was taken at 0 min and 30, 60 and 120 min after glucose was given. Blood glucose concentrations were measured by glucose oxidase method. Results are given as mean ± SEM. HFD group was considered as the control group. Figure S4 With overnight fasted, hyperinsulinemic–euglycemic clamp was conducted and lasted for 120 min. The insulin sensitivity was measured by glucose infusion rate (GIR) during the last 80 min. The glucose infusion rate in MET group was increased significantly compared with HFD group. Figure S5 Distribution of the coefficient of variation (CV value) in WAT from two run assays in LC-MS/MS was exhibited. In order to minish technical error, proteins whose CV value greater than 0.2 were excluded. Figure S6 Distribution of the coefficient of variation (CV value) in BAT from two run assays in LC-MS/MS was exhibited. In order to minish technical error, proteins whose CV value greater than 0.2 were excluded. [file 13098_2019_490_MOESM1_ESM.pptx]
